# *In situ *detection of Gag-specific CD8^+ ^cells in the GI tract of SIV infected Rhesus macaques

**DOI:** 10.1186/1742-4690-7-12

**Published:** 2010-02-16

**Authors:** Annelie Tjernlund, Jia Zhu, Kerry Laing, Kurt Diem, David McDonald, Julio Vazquez, Jianhong Cao, Claes Ohlen, M Juliana McElrath, Louis J Picker, Lawrence Corey

**Affiliations:** 1 Vaccine & Infectious Disease Institute, Fred Hutchinson Cancer Research Center, Seattle, WA, USA; 2 Departments of Medicine and Laboratory Medicine, University of Washington, Seattle, WA, USA; 3 Scientific Imaging, Fred Hutchinson Cancer Research Center, Seattle, WA, USA; 4 Immune monitoring lab, Fred Hutchinson Cancer Research Center, Seattle, WA, USA; 5 AIDS Vaccine Program, SAIC-Frederick/NCI-Frederick, Frederick, Maryland, USA; 6 Vaccine and Gene Therapy Institute, Department of Pathology, Oregon Health and Science University, Beaverton, OR, USA; 7 Vaccine and Gene Therapy Institute, Department of Molecular Microbiology, Oregon Health and Science University, Beaverton, OR, USA; 8 Vaccine and Gene Therapy Institute, Department of Immunology, Oregon Health and Science University, Beaverton, OR, USA; 9 Vaccine and Gene Therapy Institute, Oregon National Primate Research Center, Oregon Health and Science University, Beaverton, OR, USA

## Abstract

**Background:**

SIV and HIV predominantly replicate in lymphoid tissue, but the study of virus specific CD8^+ ^T cells in intact lymphoid tissue is difficult, as traditional *in situ *tetramer staining requires fresh tissue.

**Results:**

In this report, we demonstrate a novel technique using Qdot 655-conjugated peptide-MHC multimers to directly visualize SIV specific cells in cryopreserved tissue biopsies from chronically SIVmac239 infected Rhesus macaques. Qdot 655 multimers showed similar sensitivity and specificity to APC-conjugated tetramers by flow cytometry analysis, but yielded ten-fold higher signal intensity when imaged by fluorescence microscopy. Using this technique, we detected CD8^+ ^T cells which recognize an immunodominant epitope (Gag CM9) in the spleen, lymph nodes, ileum and colon. In all these tissues, the Gag CM9 positive cells were mainly located in the extra follicular T cell zone. In the ileum and colon, we found Gag CM9 positive cells concentrated in Peyer's patches and solitary lymphoid follicles, a pattern of localization not previously described.

**Conclusions:**

The use of Qdot multimers provide an anatomic and quantitative evaluation of SIV specific CD8^+ ^T cell responses in SIV pathogenesis, and may prove useful to studies of SIV specific CD8^+ ^T cell responses elicited by vaccines and other immunotherapies in the non-human primate model.

## Background

While many reports have described the pivotal role CD8^+ ^T cells play in controlling SIV and HIV-1 replication, the anatomic distribution of HIV or SIV specific CD8^+ ^T cells and their relationship to HIV/SIV infected cells has not been well characterized [1-8]. Flow cytometry analyses of virus specific CD8^+ ^T cells, identified by MHC-peptide tetramer staining, have revealed important insights into the immune cells' quantity, phenotype, and function, and the relationship between HLA type and disease progression [9,10]. However, flow cytometry does not allow direct visualization of the spatial distribution of virus specific CD8^+ ^T cells in tissue. Previous studies have demonstrated *in situ *staining of tetramers in fresh, lightly fixed, or frozen tissue using a two step enhancement methodology to visualize tetramer positive cells [11-13]. However, this technique has proven suboptimal for frozen tissue, presenting such difficulties as low signal intensity and poor cell morphology. Tetramer staining thus requires fresh tissue that should be processed within 24 h for optimal staining results and therefore does not permit the use of archived tissue samples.

We recently described a method for using Qdot 655-conjugated peptide-MHC multimers (Qdot 655 multimers) to detect HSV-2 specific cells in fresh genital skin and mucosal tissue by *in situ *staining [14]. This report describes the extension of that technique to frozen tissue samples and demonstrates that by using Qdot 655 (commercially available inherently fluorescent nanocrystals) conjugated with the Mamu-A*01 MHC Class I allele loaded with the SIVmac239 peptide Gag_181-189_CM9 (Gag CM9), it is possible to stain and detect Gag CM9 positive cells in cryopreserved lymphoid tissue from chronically SIV infected Rhesus macaques (RMs). Gag CM9 is an immunodominant cytotoxic T-lymphocyte epitope restricted by the Mamu-A*01 allele and is well characterized in the non-human primate (NHP) model, both in SIV infection and SIV vaccine models [9,15-17]

We detected Gag CM9 positive cells in spleen, lymph nodes, ileum and colon biopsies. Interestingly, in the ileum and colon, the Gag CM9 positive cells were mainly located in the inductive site of the gastrointestinal tract, e.g. Peyer's patches and solitary lymphoid follicles, respectively, a finding that to our knowledge has not been previously reported. Both Peyer's patches and solitary lymph nodes are parts of the gut associated lymphoid tissue (GALT) which is a major reservoir for SIV/HIV replication [18-23]. Thus the location of SIV/HIV specific T cells in the GALT may suggest a role for these cells in eliminating and controlling viral replication.

The availability of a sensitive and specific technique for *in situ *localization of virus specific CD8^+ ^T cells in archived samples will enable more detailed studies, including direct quantitative and anatomic assessments of the role vaccines and other immunotherapies can play in altering the CD8^+ ^T cell response in an NHP model.

## Results

### Gag CM9 Qdot 655 multimers bind to Gag CM9 specific T-cells

**Figure 1:**
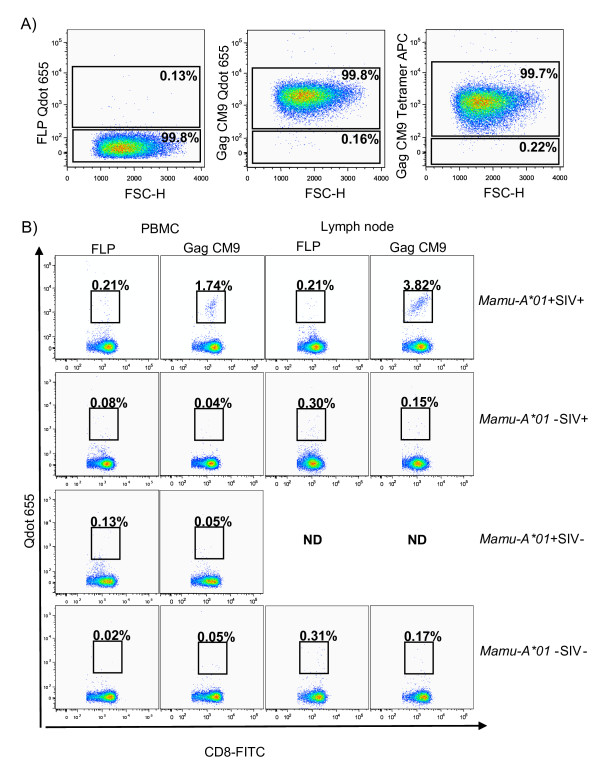
**Gag CM9 Qdot 655 multimer validation**. **A) **Flow cytometry analysis of Gag CM9 specific CD8^+ ^T cell clones showed that > 99% of the cells bound to the Gag CM9 Qdot 655 multimer or to the Gag CM9 APC tetramer. The multimer and the tetramer are coupled with the same Gag CM9 monomers. ≤ 0.13% of Gag CM9 specific cells bound to the negative control FLP Qdot 655 multimer. **B) **Flow cytometry analysis of PBMCs and single cell suspension of lymph nodes demonstrated that a distinct population of CD3^+^CD8^+ ^cells, 1.74% in blood and 3.82% in lymph node single cell suspension, from SIV infected Mamu-A*01 positive RM bound the Gag CM9 Qdot 655 multimer. ≤ 0.17% of CD3^+^CD8^+ ^cells from Mamu-A*01 positive RM that were not SIV infected or cells from Mamu-A*01 negative RM that were either SIV infected or uninfected bound the Gag CM9 Qdot 655 multimer. ≤ 0.31% of CD3^+^CD8^+ ^cells bound the FLP Qdot 655 multimer. The gating strategy was as described in Methods. ND; not done due to lack of material.

To verify the specificity of the Gag CM9 Qdot 655 multimers, we used them to stain a Gag CM9 specific T cell clone, and examined the fluorescence by flow cytometer. The T cells were stained with anti-CD3, anti-CD8 antibodies and Gag CM9 Qdot 655 multimers, or Gag CM9 APC tetramers or Qdot 655 conjugated with the Mamu-A*01 MHC Class I allele loaded with an irrelevant peptide FLP (negative control). Analysis by flow cytometry showed that all cells from the Gag CM9 T cell clone were CD3^+^CD8^+ ^cells (data not shown) and more than 99% of the cells bound Gag CM9 Qdot 655 multimers or the Gag CM9 APC tetramer (Fig. 1A). Thus, similar sensitivity was found by using flow analysis for Gag CM9 Qdot 655 multimers and the Gag CM9 APC tetramer. Less than 0.13% of the cells bound the FLP Qdot 655 multimer (negative control, Fig. 1A). Similar data were obtained with the SIV Tat_28-35 _SL8 (Tat SL8)-specific T cell clone; more than 98% of cells bound the Tat SL8 Qdot 655 multimers and ≤ 0.10% of the cells bound the FLP Qdot 655 multimer (data not shown).

To investigate if PBMCs from SIV infected Mamu-A*01 positive RMs contained SIV specific CD8^+^T cells, we stimulated the cells with Gag CM9 peptide and analyzed their ability to secrete TNF-α by intracellular cytokine staining. We found that 0.14-4.31% of CD8^+^CD69^+ ^T cells secreted TNF-α after Gag CM9 peptide stimulation and between 0.32-3.94% of CD8^+^CD69^+ ^T cells secreted TNF-α after SEB stimulation (data not shown). Next, we tested the ability of the Qdot 655 multimer to detect the Gag CM9 specific cells within this heterogeneous population of cells: we stained PBMCs from SIV infected RMs that were either Mamu-A*01 positive or Mamu-A*01 negative and PBMCs from uninfected RMs that were either Mamu-A*01 positive or Mamu-A*01 negative with Gag CM9 or FLP Qdot 655 multimers together with anti-CD3 and anti-CD8 antibodies. Flow analysis showed that 1.74-6.52% of CD3^+^CD8^+ ^cells from Mamu-A*01 positive RMs bound the Gag CM9 Qdot 655 multimer (Fig. 1B and Table 1), while ≤ 0.05% CD8^+ ^T cells from RMs that were either Mamu-A*01 negative and SIV infected, or Mamu-A*01 negative and SIV uninfected, or Mamu-A*01 positive and SIV uninfected bound the Gag CM9 Qdot 655 multimer. These percentages are similar to those previously reported using APC tetramer staining [10,24-28]. Thus, binding of the Gag CM9 Qdot 655 multimer is specific to CD8^+ ^T cells from SIV infected Mamu-A*01 positive animals and does not cross react with CD8^+ ^T cells from SIV infected, Mamu-A*01 negative animals.

**Table 1:** Percentage of Gag CM9 positive cells quantified by flow cytometry analysis of single cell suspension.

ID No	Specimen	Total counts	Live lymphocyte cell counts	CD3^**+**^CD8^**+ **^cell counts	% CD3^**+**^CD8^**+ **^of lymphocytes	Gag CM9^**+ **^cell counts	% Gag CM9 of CD3^**+**^CD8^**+**^cells
RM 1	Spleen	100 000	57 909	14 254	24.60%	1 273	8.93%
RM 2	Spleen	100 000	63 112	13 287	21.10%	1 101	8.29%
RM 3	Spleen	100 000	71 298	21 147	29.70%	2 416	11.40%

RM 1	Mesenteric LN	50 000	42 766	10 116	24.00%	509	5.03%
RM 2	Mesenteric LN	100 000	65 733	15 179	23.10%	580	3.82%
RM 3	Mesenteric LN	100 000	40 436	13 546	33.50%	701	5.17%

RM 1	PBMC	65 793	29 501	6 712	22.80%	438	6.52%
RM 2	PBMC	100 000	45 200	14 287	31.60%	249	1.74%
RM 3	PBMC	100 000	69 346	21 362	30.80%	776	3.63%

The total cell count, live lymphocyte cell count, CD3^+^CD8^+ ^cell counts, percentage of CD3^+^CD8^+ ^cells in the live lymphocyte population, Gag CM9^+ ^cell counts and percentage of Gag CM9^+ ^cell in the CD3^+^CD8^+ ^population was calculated by essaying flow staining on single cell suspension of spleen, mesenteric lymph nodes and PBMCs. Cells were gated on live CD3^+^CD8^+ ^cells. LN; lymph node

We also evaluated cell suspensions of spleen and lymph node from Mamu-A*01 positive, SIV infected RMs; 8.29-11.40% and 3.82-5.17% of the CD3^+^CD8^+ ^T cells, respectively, bound the Gag CM9 Qdot 655 multimer (Fig. 1B and Table 1). ≤ 0.17% CD8^+^T cells from Mamu-A*01 negative, SIV infected RMs or from Mamu-A*01 positive, SIV negative RMs bound the Gag CM9 Qdot 655 multimer (Fig. 1B). ≤ 0.31% of the CD8^+ ^T cells of any of the single cell suspensions described above bound to the Qdot 655 multimer loaded with the negative control peptide FLP, verifying that nonspecific binding of the Qdot 655 multimer is low.

### Staining pattern and staining intensity of Gag CM9 Qdot 655 multimer positive cells

**Figure 2:**
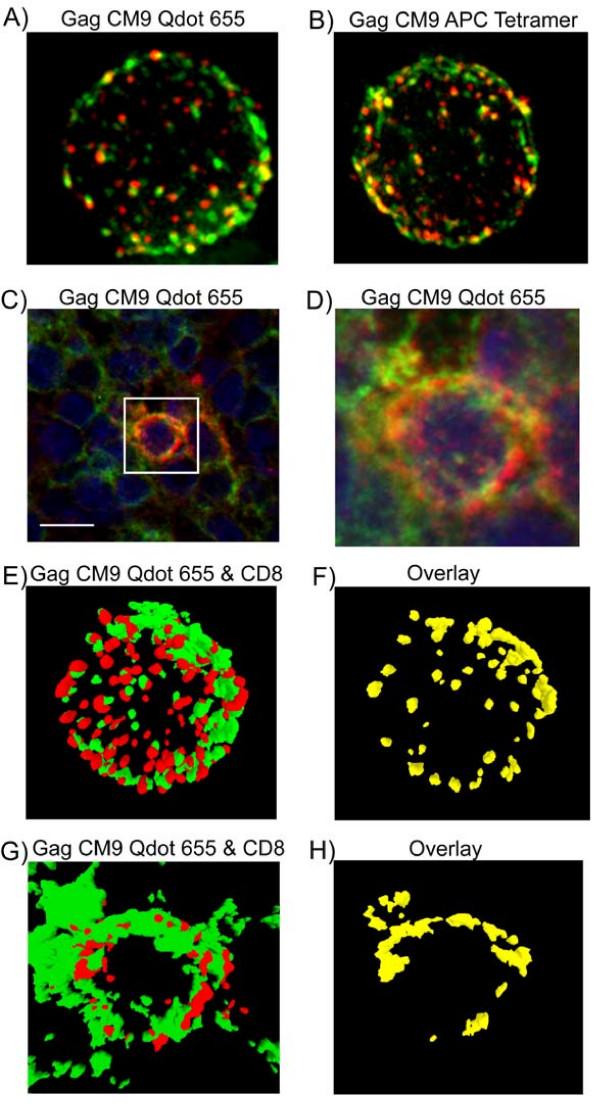
**Staining Patterns of Gag CM9**. **A) **Fluorescence image of a lymphocyte from an SIV infected Mamu-A*01 positive RM stained with Gag CM9 Qdot 655 multimer (red) and CD8 (green). **B) **Fluorescence image of a lymphocyte from an SIV infected Mamu-A*01 positive RM stained with Gag CM9 APC Tetramer (red) and CD8 (green). **C) **Confocal fluorescence image of a colon tissue section from an SIV infected Mamu-A*01 positive RM stained with Gag CM9 Qdot 655 multimer (Red), CD8 (green) and Dapi (blue). Scale bar = 10 μm. **D) **A magnified view of the region indicated in panel **C**. Cells stained with Qdot 655 multimer or APC tetramer show a punctate Gag CM9 staining pattern. All images were acquired with a 100×/1.4 oil immersion objective and further deconvolved. **E-H) **Volocity (Improvision) software was used to generate a surface model of the CD8^+^Gag CM9^+ ^cells shown in panel **A **(**E, F) **and panel **D **(**G**,**H)**. **E, G: **CD8 (green), Gag CM9 Qdot 655 multimer (red); **F, H: **overlap (yellow) of CD8 and Gag CM9 Qdot 655 multimer staining.

Confocal microscopy revealed a punctate staining pattern of individual cells stained with the Gag CM9 Qdot 655 multimers (Fig. 2), as has been previously reported for tetramer staining [11,12]. We observed this punctate pattern in the Gag CM9 T cell clone (data not shown), Gag CM9 Qdot 655 multimer specific CD8^+ ^T cells from lymph node single cell suspensions (Fig. 2A), and Gag CM9 Qdot 655 multimer specific CD8^+ ^T cells in colon tissue biopsies (Fig. 2C and 2D). Similar staining patterns were found using the Gag CM9 APC tetramer with single cell suspensions of lymph nodes (Fig. 2B). Detailed 3-D modeling of the staining pattern using Volocity (Improvision) software revealed the close proximity between CD8 molecules and the T cell receptor (Fig. 2E, G). The CD8 staining and the Gag CM9 staining pattern overlapped almost entirely (Fig. 2F, H).

**Figure 3:**
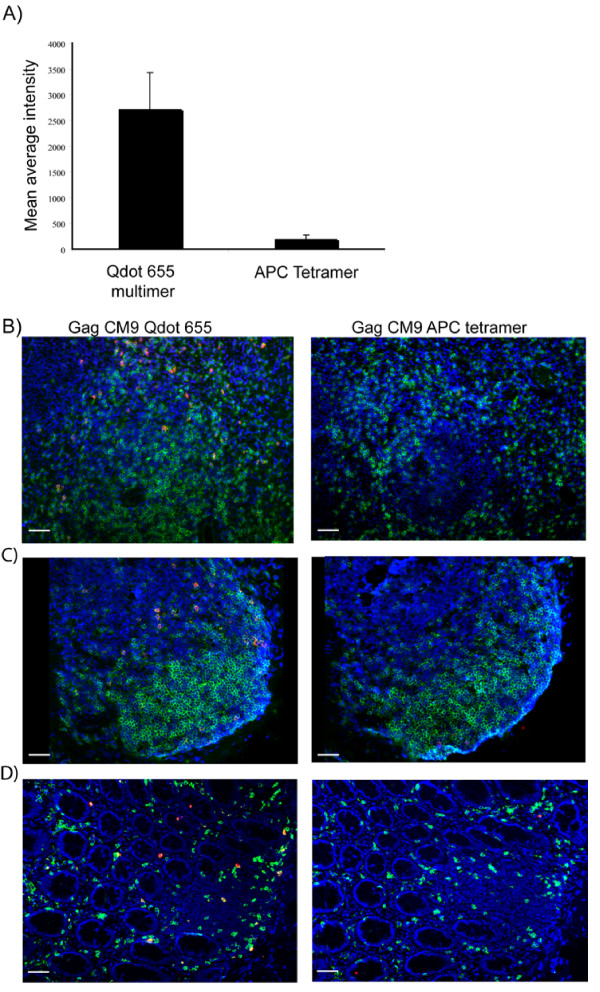
**Intensity comparison between Gag CM9 Qdot 655 multimer and Gag CM9 APC-tetramer**. **A) **Single cell suspension cells of a lymph node from an SIV infected Mamu-A*01 positive RM were stained with Gag CM9 Qdot 655 multimer or with Gag CM9 APC tetramer and the staining intensity was measured. Z stack average intensity projections for six cells was used and the mean average intensity was calculated by using Image J Software. A ten-fold higher mean average intensity was found for Gag CM9 Qdot 655 multimer as compared to the Gag CM9 APC tetramer, even though the same monomers are used in each case. **B-D) **Fluorescence images of tissue sections from an SIV infected Mamu-A*01 positive RM stained with CD8 in green, Gag CM9 Qdot 655 multimer in red (left column) or Gag CM9 APC Tetramer in red (right column) and dapi in blue. **B) **Images of spleen sections stained with Gag CM9 Qdot 655 multimer demonstrated abundant Gag CM9 specific cells (left column) whereas the consecutive section stained with Gag CM9 APC Tetramer showed no Gag CM9 positive cells (right column). **C) **Gag CM9 positive cells were detected in a solitary lymphoid follicle within the colon section (right column) when Gag CM9 Qdot 655 multimer was used, whereas when the consecutive slide was stained with Gag CM9 APC Tetramer no Gag CM9 positive cells could be detected (left column). **D) **Gag CM9 positive cells were detected both in the lamina propria and in a small solitary lymph node in the colon (right column) when stained with Gag CM9 Qdot 655 multimer but when the consecutive slide was stained with Gag CM9 APC tetramer no Gag CM9 positive cells were detected (left column). Images were collected with a 20×/0.75 objective. Scale bar = 50 μm.

Cells stained with the Gag CM9 APC tetramer needed longer exposures than those stained with the Gag CM9 Qdot655 multimer to be visualized by fluorescence microscopy. We performed intensity measurements of Z plane projections of cells stained with the Gag CM9 Qdot655 multimer or the Gag CM9 APC tetramer. A ten-fold higher mean average staining intensity was found for cells stained with the Gag CM9 Qdot655 multimer as compared to cells stained with the Gag CM9 APC tetramer (Fig. 3A). In tissue biopsies, we were not able to detect any Gag CM9 positive cells using the Gag CM9 APC tetramer for *in situ *staining of spleen (Fig. 3B), lymph node (data not shown), ileum (data not shown) or colon (Fig. 3C-D) tissue sections, even when samples were exposed for ten times longer than the biopsies stained with Gag CM9 Qdot 655 multimer.

### Percentage of Gag CM9 Qdot 655 multimer positive cells quantified by *in situ* staining

**Figure 4:**
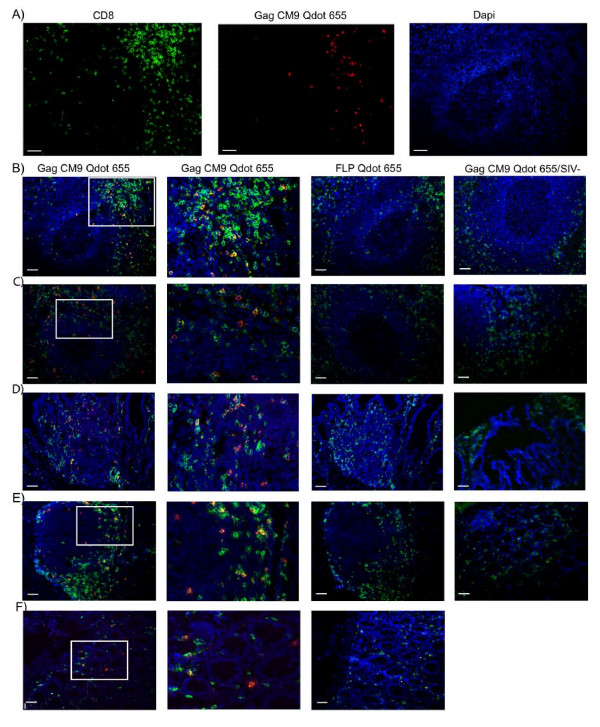
***In situ *staining of CD8^+^Gag CM9^+ ^cells in frozen tissue**. **A) **Fluorescence images of a spleen tissue section from an SIV infected Mamu-A*01 positive RM stained with CD8 (green), Gag CM9 or FLP Qdot 655 multimer (red), and DAPI (blue) demonstrating that the Gag CM9 positive cells are CD8^+^. Fluorescence images of **B) **spleen sections, **C) **mesenteric lymph node sections, **D) **ileum sections with peyer's patch, **E) **solitary lymph node in colon and **F) **images of lamina propria in colon tissue from SIV infected Mamu-A*01 positive RM. Left-most column, second column, and right most column show Gag CM9 Qdot 655 multimer (red). The second column is a magnification of the left column images. Third column; FLP Qdot 655 multimer (red). Right-most column, similar sections from a non-infected Mamu-A*01 negative RM. Images were collected with a 20×/0.75 objective. Scale bar = 50 μm.

Snap frozen biopsies (spleen, lymph nodes, colon and ileum) from chronically SIV infected Mamu-A*01 positive RMs were stained with Gag CM9 Qdot 655 multimers followed by addition of anti-CD8 antibody. CD8 staining was not performed in tandem with Qdot or tetramer staining, as some anti-CD8 antibodies may interfere with or enhance tetramer binding to the TCR ligand [12,29]. Double staining with Gag CM9 Qdot 655 multimer and CD8 confirmed that Gag CM9 positive cells were CD8^+ ^(Fig. 4A).

Gag CM9 positive cells were detected in all of the frozen tissues analyzed that were from chronically SIV infected and Mamu-A*01 positive RMs (Fig. 4), including those tissue sections with low SIV copy numbers (Table 2). The percentage of Gag CM9 specific CD8^+ ^cells in all tissues analyzed ranged from 2.43%-9.59% (Table 3), with some variation between different lymphoid compartments. In the spleen (Fig. 4A and 4B), 6.80%- 9.59% of the CD8^+ ^T cells were specific for the Gag CM9 Qdot 655 multimers; in the submandibular lymph node, 3.30%- 5.21%; and in mesenteric lymph nodes (Fig. 4C), 3.26%- 6.51%. In the ileum (Fig. 4D), 2.43%- 2.97% of the CD8^+ ^T cells were specific for the Gag CM9 Qdot 655 multimers; and in the colon (Fig. 4E and 4F), 3.10%- 6.74%. Thus, the highest percentage of Gag CM9 positive cells were found in the spleen; the colon, mesenteric-, and submandibular lymph nodes had similar ranges of Gag CM9 positive cells; and the ileum had the lowest percentages of Gag CM9 positive cells of all lymphoid tissues analyzed. Because the ileum and colon contain lamina propria with a less dense cell population than in Peyer's patches and solitary lymph nodes, a higher variability in total cell number was found in these tissues than in the other tissue types analyzed.

**Table 2:** SIV DNA and RNA quantification.

ID No	Specimen	SIV RNA copy eq/mL	SIV DNA copies/10,000 cells	SIV RNA copies/250 ng total RNA
RM 1	Plasma	50		
RM 2	Plasma	3.3 × 10^6^		
RM 3	Plasma	< 30		

RM 1	Spleen		0	0
RM 2	Spleen		142	15986
RM 3	Spleen		0	0

RM 1	Submandibular LN		9	120
RM 2	Submandibular LN		391	29155
RM 3	Submandibular LN		19	28

RM 1	Mesenteric LN		10	7
RM 2	Mesenteric LN		283	9736
RM 3	Mesenteric LN		18	324

RM 1	Ileum		ND	ND
RM 2	Ileum		14	74
RM 3	Ileum		0	0

RM 1	Colon		0	0
RM 2	Colon		48	18755
RM 3	Colon		0	0

Plasma SIV RNA was assessed using a real-time RT-PCR assay and SIV RNA and DNA from lymphoid tissue biopsies were assayed by RT-PCR and PCR. Biopsy RNA and DNA were amplified with the Rhesus Monkey GAPDH kit (Applied Biosystems Inc) to confirm the nucleic acids were amplifiable (data not shown). LN; lymph node and ND; not done due to lack of material.

**Table 3:** Percentage of Gag CM9 positive cells quantified by imaging analysis of *in situ *stained lymphoid tissue sections.

ID No	Specimen	Total cells/mm^**2**^	CD8^**+ **^cells/mm^**2**^	Gag CM9^**+ **^cells/mm^**2**^	% Gag CM9 of CD8^**+ **^cells
RM 1	Spleen	16 678 (± 2 032)	2 399 (± 386)	164 (± 39)	6.80 (± 0.6)
RM 2	Spleen	20 789 (± 3 552)	2 048 (± 627)	198 (± 70)	9.59 (± 2.0)
RM 3	Spleen	20 276(± 3 877)	2 694 (± 494)	206 (± 49)	7.78 (± 2.0)

RM 1	Submandibular LN	31 340 (± 4 076)	3 333 (± 511)	170 (± 11)	5.21 (± 1.1)
RM 2	Submandibular LN	20 789 (± 3 552)	4 885 (± 1 134)	255 (± 78)	5.16 (± 0.6)
RM 3	Submandibular LN	18 754 (± 3 317)	3 684 (± 232)	122 (± 22)	3.31 (± 0.5)

RM 1	Mesenteric LN	23 330 (± 3 458)	6 876 (± 1 225)	262 (± 26)	3.91 (± 1.0)
RM 2	Mesenteric LN	17 975 (± 2 866)	2 969 (± 685)	183 (± 48)	6.51 (± 2.8)
RM 3	Mesenteric LN	21 588(± 3 470)	6 476 (± 1 163)	200 (± 42)	3.26 (± 1.4)

RM 1	Ileum	ND	ND	ND	ND
RM 2	Ileum	6 812 (± 2 603)	701 (± 7)	21 (± 3)	2.97 (± 0.4)
RM 3	Ileum	6 793 (± 1 246)	629 (± 206)	15 (± 5)	2.43 (± 0.3)

RM 1	Colon	10 049 (± 4 193)	1 145 (± 135)	77 (± 16)	6.74 (± 0.9)
RM 2	Colon	11 814 (± 5 130)	2 287 (± 1 424)	76 (± 52)	3.10 (± 0.5)
RM 3	Colon	11 126 (± 4 207)	888 (± 160)	42 (± 9)	4.80 (± 0.9

The average total cell number/mm^2^, CD8^+ ^cells/mm^2^, Gag CM9^+ ^cells/mm^2 ^and percentage of Gag CM9 Qdot 655 multimer positive cells in the CD8^+ ^cell population was calculated by using the image J particle counting program. The standard deviation is shown in brackets. Staining was performed at least three times for each specimen and a total tissue area of 4.6 × 10^6 ^μm^2 ^was counted for each specimen. LN; lymph node and ND; not done due to lack of material.

We also stained the tissue biopsies with Qdot 655 multimers containing peptides corresponding to the following known MamuA*01 restricted SIV epitopes (for full description see Table 4): Gag LW9, Gag QI9, Gag LF8, Pol LV10, Pol QV9, Pol SV9, Env CL9, Env ST10, Env TL9, Tat SL8, or VIF QA9, or FLP peptides. Few cells (< 0.01%) were positive for Gag LW9, Gag QI9, Gag LF8, or Pol SV9 in the spleen and no positive cells were detected for Pol LV10, Pol QV9, Env CL9, Env ST10, Env TL9, Tat SL8, or VIF QA9, consistent with previous reports that the Gag CM9 response is dominant in chronically SIV infected Mamu-A*01 positive RMs [16,17]. To confirm specificity of our Qdot 655 multimer staining, we used the same Qdot 655 conjugated with the Mamu-A*01 MHC Class I allele but loaded with an irrelevant peptide (FLP) as a negative control. No staining was seen with the FLP Qdot 655 multimer (Fig. 4B-F, third column). Cryopreserved spleen, mesenteric lymph nodes, ileum and colon tissues biopsies were obtained from non SIV infected Mamu-A*01 negative RM and used as further negative controls. They were stained with the Gag CM9 Qdot 655 multimer (Fig. 4B-E, right column) and with the FLP Qdot 655 multimer (data not shown); no positive cells were detected. We found that the intraepithelial cells in the ileum and in the colon showed higher autoflorescence than cells in Peyer's patches, solitary lymphoid follicles, lymphoid follicles and spleen; and hence careful analysis of low frequency cells, particularly in the intraepithelial region, is of importance to account for this background fluorescence.

**Table 4:** Nomenclature of Mamu-A*01-restricted epitopes.

Protein	Amino acid positions	Sequence	Short name
SIV Gag	149-157	LSPRTLNAW	Gag LW9
SIV Gag	181-189	CTPYDINQM	Gag CM9
SIV Gag	245-262	QNPIPVGNI	Gag QI9
SIV Gag	372-379	LAPVPIPF	Gag LF8
SIV Pol	147-156	LGPHYTPKIV	Pol LV10
SIV Pol	592-600	QVPKFHLPV	Pol QV9
SIV Pol	625-633	STPPLVRLV	Pol SV9
SIV Env	233-241	CAPPGYALL	Env CL9
SIV Env	620-628	TVPWPNASL	Env TL9
SIV Env	726-735	SSPPSYFQQT	Env ST10
SIV Tat	28-35	STPESANL	Tat SL8
SIV Vif	144-152	QVPSLQYLA	Vif QA9

### Spatial distribution of Gag CM9 positive cells in lymphoid tissue

**Figure 5:**
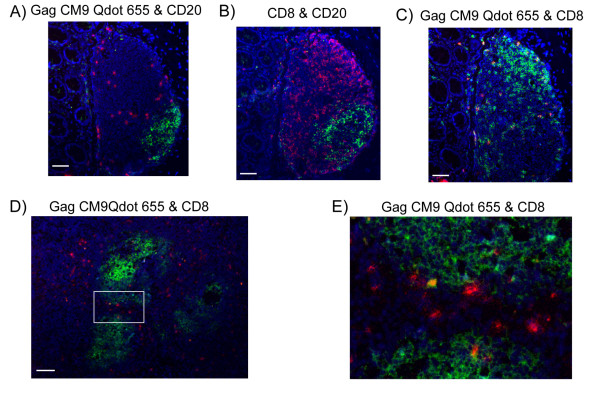
**Gag CM9 T cells are located in the extra follicular area of lymphoid tissue**. Fluorescence images showing sections from an SIV infected Mamu-A*01 positive RM. **A-C) **Images of a solitary lymphoid follicle in a colon section. **A) **Gag CM9 positive cells are located in the extra follicular region. Gag CM9 Qdot 655 multimer (red), CD20 (green). **B) **No CD8^+ ^cells were located in the extra follicular region. CD8 (red), CD20 (green). **C) **All CM9 positive cells were CD8^+^. Gag CM9 Qdot 655 multimer (red), CD8 (green). **D) **Image of a mesenteric lymph node section showing that the majority of the Gag CM9 positive cells are located in the extra follicular region, but some are in the junction of the extra follicular and follicular area. Gag CM9 Qdot 655 multimer (red), CD20 (green) **E) **A magnified view of the region indicated in panel **D **showing the Gag CM9 positive cells the junction of the extra follicular and follicular area. All sections were stained with DAPI (blue). Images were collected with a 20×/0.75 objective. Scale bar = 50 μm.

Gag CM9 positive cells were abundant and widely dispersed throughout the T cell zone in all the tissues analyzed (Fig. 4). The cells showed a clustered staining pattern (Fig. 4 and 5D), indicating possible clonal expansion. In the ileum and colon the Gag CM9 positive cells were mainly located in Peyer's patches and solitary lymph nodes, respectively (Fig. 4D and 4E), with few Gag CM9 positive cells dispersed in the lamina propria (Fig. 4F).

We next stained for CD20 to localize the lymphoid follicles, which harbor HIV infected CD4 cells, and follicular dendritic cells, which contain infectious virus particles [21,30,31]. Co-staining of CD20 and Gag CM9 Qdot 655 multimer revealed that the majority of the Gag CM9 positive cells in spleen, lymph node, ileum and colon were excluded from the lymphoid follicles (Fig. 5). However, some Gag CM9 positive cells were seen in the junction between the follicle and the extra follicular area, and in the extra follicular area (Fig. 5D and 5E), indicating that some of these cells are able to enter the B cell follicle and therefore have the potential to come in close proximity with infected cells or cells carrying SIV particles.

## Discussion

We have developed a method to allow direct visualization of virus specific cells in frozen tissue. The use of SIV specific APC tetramers for *in situ *staining traditionally requires a two-step enhancement methodology and the use of fresh tissue, as staining in frozen tissue results in low signal to noise ratio [11-13]. In this paper, we report technical improvements in staining frozen tissues using commercially available Qdots (nanocrystals). Qdots have an intrinsic brightness and are constructed to have seven to eight streptavidin molecules covalently attached to each Qdot particle and thus are able to bind 32 peptide-MHC monomers under saturated conditions. The enhanced binding and brightness are the likely explanation for our ability to detect virus specific cells even in frozen tissue. Imaging analysis of the Gag CM9 Qdot 655 multimer demonstrated a tenfold higher mean staining intensity than the Gag CM9 APC tetramer, even though similar sensitivity and specificity was found for the two different compounds during flow cytometry analysis. Furthermore, the frequency of the Gag CM9 Qdot 655 multimer positive cells that were detected by *in situ *staining in blood, spleen, and lymph nodes was similar to that detected by flow cytometry analysis. Thus, the Qdot 655 multimer, when used with our optimized protocol on cryopreserved tissue, allows a more detailed *in situ *analysis of Gag CM9 specific CD8^+ ^T cells, and provides the technology for monitoring T cell responses during SIV and other viral infections.

Our *in situ *study demonstrates detection of Gag CM9 positive cells in frozen lymphoid tissue (spleen, lymph nodes and gastrointestinal tract) analyzed from chronically SIVmac239 infected Mamu-A*01 positive RMs. The Gag CM9 positive cells were abundant, ranging from 2.43%- 9.59% of all CD8^+ ^cells, confirming reports using flow cytometry or *in situ *staining of fresh tissues using tetramers [10,24-28]. We also looked for CD8^+ ^T cells recognizing other Mamu-A*01 restricted epitopes. Specifically, we did not detect any Tat SL8 (an epitope that is immundominant in early SIV infection) specific CD8^+ ^T cells in our tissue sections, which is most likely due to the fact that these biopsies are taken from chronically infected rhesus macaques (77-85 days post-SIV infection), and the Tat SL8 response usually escapes during the acute infection phase [17]. Furthermore, no subdominant Mamu-A*01 restricted SIV CD8^+ ^T cells were detected, confirming that the Gag CM9 response is dominant in chronically SIV infected Mamu-A*01 positive RMs [17].

Among the tissue types analyzed, the highest proportion of Gag CM9 cells was detected in the spleen, consistent with previous findings [24,28]. Some studies have found HIV and SIV specific cells to be more abundant in lymphoid tissue and in the female reproductive tract than in peripheral blood, while others have shown no such differences [24-28,32,33]. In this study we found some variability between the different lymphoid compartments. Although our current study did not analyze the Gag CM9 response in tissue from the female reproductive tract, we did see abundant Gag CM9 positive cells in the colon. Since the female reproductive tract and the colon are the port of entry for sexual transmission of HIV/SIV it is most likely important to have HIV- or SIV- specific CD8^+ ^T cells in these locations to have the potential to control the infection at its initial site.

In two of the three RMs, a similar percentage of Gag CM9 positive cells was found in PBMCs as in lymphoid tissue, while the third RM had a lower percentage of Gag CM9 positive cells in PBMCs as compared to lymphoid tissue. We found no correlation between viral load and the number of Gag CM9 positive cells/mm^2 ^or the percentage of Gag CM9 positive cells in the biopsies analyzed; however, this may be due to the small sample size of animals.

We found Gag CM9 positive cells widely dispersed throughout the T cell zone in all the lymphoid tissues analyzed. Of interest, we detected clusters of Gag CM9 cells that may be indicative of recent clonal expansion of these cells. To our knowledge, we are the first to show accumulation of Gag CM9 positive cells in Peyer's patch and in solitary lymphoid follicles in ileum and colon, respectively. We also detected Gag CM9 positive cells in the lamina propria (effector site), but to a smaller extent than in the Peyer's Patch and in solitary lymph nodes. Both of these anatomical sites are a part of the GALT, which is considered to comprise most of the secondary lymphoid organ system and harbors the largest number of recently activated memory CD4^+ ^T cells [18,20]. The GALT is one of the largest reservoirs for SIV/HIV replication [19-23], and CD4^+ ^T cells are massively depleted there during early infection [18,20,23,34]. It is therefore crucial that virus specific cells are present in these sites to mount a successful immune response against the virus.

CD20 staining was used to visualize the follicular area of the lymphoid tissue. It has been reported that HIV infected CD4^+ ^cells and follicular dendritic cells harboring infectious virus particles persist in lymphoid follicles [21,30,31]. While the majority of the Gag CM9 positive cells were detected in the extra follicular area, some were observed in the border between the follicle and the extra follicular area or in the follicular area, confirming findings from previous studies of HIV infected individuals and SIV infected monkeys [30,35]. Hong *et al*. recently showed that in SIV infected RMs a small number of the Gag CM9 tetramer positive cells that were located near or within a lymphoid follicle had a CD8^low ^profile, and hypothesized that the CD8^low ^profile was due to either T cell receptor signaling or low levels of IL-7 in the B cell follicle [35]. Another study showed that a subset of CD8^+ ^T cells from uninfected humans home to the lymphoid follicles in a CXCR5-dependent manner, and that the cells in this location have characteristics of a non-cytolytic effector memory phenotype [36]. Together with these previous observations, our findings suggest that some CD8^+ ^T cells are able to enter the lymphoid follicle. It would be of interest to further explore the role of these virus specific CD8^+^cells located near or within the B cell follicle, to understand both the immunological interactions between the different cells (CD8^+ ^T cells, CD4^+ ^T cells, B cells and follicular dentritic cells) within this compartment and the CD8^+ ^T cells' role in controlling SIV replication. The Qdot 655 multimer may prove useful in undertaking a detailed *in situ *analysis of the CD8^+ ^T cell responses in SIV infection.

## Conclusion

In this study we demonstrate that by using the Gag CM9 Qdot655 multimer instead of Gag CM9 APC tetramers it is possible to directly visualize virus specific CD8^+ ^T cells in cryopreserved lymphoid tissue biopsies. Qdot 655 multimers were found to have similar sensitivity and specificity as APC-conjugated tetramers by flow cytometry analysis, but yielded ten-fold higher signal intensity when imaged by fluorescence microscopy. Using this technique, we detected Gag CM9 specific CD8^+ ^T cells in spleen, lymph nodes, ileum and colon. In the ileum and colon, we found Gag CM9 positive cells concentrated in Peyer's patches and solitary lymphoid follicles; a pattern of localization not previously described. The availability of a sensitive and specific technique for *in situ *localization of virus specific CD8^+ ^T cells may prove useful in the study of the pathogenesis of SIV infection and the role vaccines and immunotherapy may play in altering the CD8^+ ^T cell response in the NHP models.

## Methods

### Animals and virus

Six purpose-bred RMs (*Macaca mulatta*) of Indian genetic background were used in this study. Tissue biopsies, single cell suspensions, and PBMCs were obtained from three Mamu-A*01 positive RMs that were chronically infected with SIVmac239 and from one Mamu-A*01 negative, SIV negative RM. PBMCs were also obtained from one Mamu-A*01 positive, SIV negative RM and one Mamu-A*01 negative RM chronically infected with SIVmac239. SIVmac239 infections were initiated with intravenous injection of 5 ng equivalents of SIV p27. The RMs were housed at the Oregon and Washington National Primate Research Centers in accordance with standards of the Center's Animal Care and Use Committee and the NIH "Guide for the Care and Use of Laboratory Animals" [37]. Animal experiments at both institutions were approved by the National Primate Research Center's Animal Care and Use Committee.

### Specimen collection

Submandibular and mesenteric lymph nodes, spleen, ileum and colon biopsies were obtained at necropsy, (77-85 days post-SIV infection). The biopsies were snap frozen in OCT media (Sakura Finetek USA Inc. Torrance, CA) and kept at -80°C until sectioning. Single-cell suspensions of lymph node cells and splenocytes were obtained by mechanically disaggregating the tissues, which were filtered through a mesh followed by standard Ficoll-Hypaque separation. PBMCs were isolated by standard Ficoll-Hypaque centrifugation from whole blood. The single cell suspensions were stored in freezing medium (10% DMSO in fetal bovine serum (FBS)) at -195°C.

### Viral quantification

Plasma SIV RNA was assessed using a real-time RT-PCR assay [38] with a threshold sensitivity of 30 SIV Gag RNA copy equivalents per milliliter of plasma.

SIV DNA from the cryopreserved tissue biopsies was isolated using the QIAamp DNA Mini Kit (Qiagen, Valencia CA). SIV RNA from the cryopreserved tissue biopsies was isolated using the RNAgents Total RNA System (Promega, Madison WI). Both were quantified using a Nanodrop spectrophotometer (Nanodrop Technologies Wilmington, DE). The amount of DNA assayed per reaction was equivalent to 10,000 cells assuming 6.6 pg DNA per cell. The amount of RNA assayed was 250 ng per reaction. A SIVmac239 Gag sequence was cloned into the pCR 2.1 TOPO vector (Invitrogen, Eugene, OR) and RNA was made from this plasmid using the MEGAshortscript kit (Ambion, Applied Biosystems Inc, Foster City, CA). The RNA was quantified using a Nanodrop spectrophotometer (Nanodrop Technologies), and diluted to 30,000 RNA copies/μL. This standard was used to quantify SIV RNA in a ABI Prism 7700 machine using the Invitrogen Ultrasensitive Kit. The two amplification primers were SHIVKU1F (AGG CTG CAG ATT GGG ACT TG) and SHIVKU1R (CCC TAA GTT GTC CTT GTT GTG GA) and the probe was SHIVKU-1 (6 FAM AGC ACC CAC ACC CAG MGB). The PCR thermocycler conditions were set for 50°C for 15 min for cDNA synthesis, 95°C for 2 m for denaturation; and 42 cycles of amplification at 95°C for 15 s, and 60°C for 30 s. For the DNA assay, DNA from the 3D8 cell line [34] was prepared using the same Qiagen kit as before and diluted to 50,000 copies/μL. This standard was used to quantify SIV DNA in the same thermocycler as before using the ABI Taqgold enzyme system. The PCR thermocycler conditions were 95°C for 10 min followed by 42 cycles at the same temperatures and times as above.

### Epitope-specific T cell clones

Gag CM9-specific and Tat SL8-specific CD8^+ ^T cell clones were produced as previously described [39]. Briefly, PBMCs were isolated from a chronically SIVmac239 infected Mamu-A*01 positive RM and the cells were stimulated with irradiated autologous PBMC pulsed with Gag CM9 or Tat SL8 peptide (SynPep Corp., Dublin, CA) [40]. After 2 rounds of restimulation, the Gag CM9- or Tat SL8-specific CD8^+ ^T cell were cloned by limiting dilution. Positive wells were tested for antigen specificity by flow cytometry using intracellular cytokine staining for IFN-γ production and clonality was confirmed by staining with Gag CM9 (Beckman Coulter, Miami, FL) or Tat SL8 (NIH AIDS Research and Reference Reagent Program) peptide/MHC-tetramers. The Gag CM9- and Tat SL8-specific CD8^+ ^T cell clones were immortalized by transduction with a human telomerase reverse trancriptase/nerve growth factor receptor (hTERT/NGFR) construct and the transduction efficiency was determined by surface staining for NGFR expression using anti-human NGFR-PE (clone C40-1457; BD/PharMingen, San Diego, CA). Transduced cells were further enriched by surface staining with anti-human NGFR-PE followed by PE microbead selection according to manufacturer's recommendation (Miltenyi, Auburn, CA).

### Synthesis of Qdot 655 multimers and APC tetramers

Qdot 655-conjugated peptide-MHC multimers were formed *in vitro *as previously described [14]. Briefly, biotinylated Mamu-A*01/β_2_m/peptide monomers were produced with the known Mamu-A*01-restricted epitopes as outlined in Table 4 [40]. A peptide (FLPSDYFPSV (FLP)) from the core protein of Hepatitis B was also generated for use as a negative control. Streptavidin-coated APC (Prozyme, San Leandro, CA) or Qdot 655 (Invitrogen) was conjugated with a saturating amount of biotinylated Mamu-A*01/β_2_m/peptide monomers.

### Cell surface flow cytometry staining

PBMC or single cell suspensions from spleen and lymph nodes were resuspended, washed in PBS, and stained with LIVE/DEAD Fixable Violet Dead Cell Stain (Invitrogen) for 30 min at room temperature (RT). The cells were then washed in PBS with 2% FBS followed by incubation with Gag CM9 Qdot 655 multimer or with FLP Qdot 655 multimer for 30 min at RT. Anti-CD8-FITC (SK1) and anti-CD3-PerCP (SP34-2) antibodies (BD biosciences) were added and cells were incubated for an additional 20 min at RT followed by washing in PBS with 2% FBS. The cells were fixed for 10 min in 2% formaldehyde and cell surface expression was assessed using a LSR II cytometer (LSR II; Becton Dickinson). All FACS analyses were performed using FlowJo^® ^software (Treestar, Inc; OR). Single cells were selected and dead cells excluded. CD3^+^CD8^+ ^cells were then selected and observed for Qdot 655 or APC staining.

### Intracellular TNF-a staining

SIV-specific CD8^+ ^T cell responses were measured by flow cytometric intracellular cytokine analysis of PBMCs as previously described [41]. Briefly, PBMC (1 × 10^6^) were incubated at 37°C for 1 h with co-stimulatory antibodies against CD28 and CD49d (0.5 μg of each antibody; (BD Biosciences) and Gag CM9 peptide (2 μg peptide/sample) to stimulate cognate SIV-specific CD8^+ ^T cells. Staphylococcal enterotoxin B (SEB 0.2 μg/ml; Toxin Techonology, Sarasota, FL) was used as a positive control and co-stimulation in the absence of antigen served as a negative-control. Cells were then treated with 10 μg of brefeldin A (10 μg/ml; Sigma) to inhibit protein trafficking for an additional 5 h at 37°C. Cells were then washed with PBS plus 2% FBS followed by surface and intracellular staining with the following conjugated antibodies from BD Biosciences: CD3 (SP34-2), CD4 (L200), CD8 (SK1), CD69 (L78) and TNF-α (6401.1111). Flowcytometric analysis was performed on an LSR II cytometer (Becton Dickinson). The Gag CM9 and the SEB data were corrected against the negative control.

### *In Situ* staining

7 μm thick sections from cryopreserved biopsies were cut using a cryostat, mounted on super frost plus slides (Fisher Scientific, Pittsburg, PA) and washed in PBS with 0.1% BSA. The sections were blocked in PBS containing 2% BSA and 5% normal human serum for 30 min at RT, followed by incubation with Qdot 655 multimers over night at +4°C. The next day the sections were washed in PBS with 0.1% BSA and fixed in 2% formaldehyde for 10 min followed by a wash in PBS with 0.1% saponin. The sections were incubated with mouse anti-CD8 (RPA-T8) or mouse anti-CD20 (2H7) antibodies (BD Biosciences) for 1 h at RT, then washed in PBS with 0.1% saponin and incubated with donkey anti-mouse *IgG *Alexa Fluor 488 (Invitrogen) for 30 min at RT. The sections were washed in PBS with 0.1% saponin followed by a wash in PBS prior to incubation with DAPI (Fluka, Sigma-Aldrich, St Louis, MO) and a final wash in MilliQ water. The tissue sections were mounted in Mowiol 40-88 containing 2.5% wt/vol DABCO (Sigma-Aldrich).

### Detection and quantification of Qdot 655 conjugated pMHC multimers

Fluorescence microscopy (Nikon Eclipse TE 2000-S; Nikon Instruments Inc., Melville, NY) was used to visualize CD8^+ ^cells and CD20^+ ^cells as well as cells positive for Qdot 655 or APC tetramer in the stained tissue sections. Each biopsy was cut and stained for CD8, or CD20 with the Qdot 655 multimers or the Gag CM9 APC tetramer, and counterstained with DAPI. At least three sections were prepared for each biopsy, and five fields were analyzed for each tissue section. The cells were enumerated in tissue fields of 641.5 × 479.3 micrometers and a total tissue area of 4.6 × 10^6 ^μm^2 ^was analyzed per biopsy. At least 2,000 CD8^+ ^cells for each section of spleen and lymph nodes, 900 CD8^+ ^cells for each section of the colon, and 600 CD8^+ ^cells for each section of the ileum were counted using the particle counting algorithm of public domain imaging software ImageJ. Total cells/mm^2^, CD8^+ ^cells/mm^2^, Gag CM9 positive cells/mm^2 ^and the percentage of Gag CM9 positive in the CD8^+ ^cell population was calculated.

To evaluate the staining pattern of CD8 and Gag CM9 positive cells, high resolution three dimensional images of the tissue sections were acquired either by confocal microscopy on a Zeiss LSM 510 laser scanning confocal microscope (Carl Zeiss MicroImaging, Inc., Thornwood, NY), or by deconvolution microscopy on a DeltaVision RT microscope (Applied Prescision Inc., Issaquah, WA). Excitation lasers, detection bands and beam splitter set-up for the confocal microscopy were as previously described [14]. Volocity software (Perkin Elmer, Waltham, MA) was used for detailed 3-D modeling of the staining patterns. Average intensity Z plane projections for single cell suspension cells stained with Gag CM9 Qdot 655 multimer or Gag CM9 APC tetramer were used for intensity calculation by Image J software.

## Competing interests

The authors declare that they have no competing interests.

## Authors' contributions

AT and JZ developed the *in situ *staining in frozen tissue technology. AT designed and conducted the experiments, prepared the figures and the manuscript and provided funding. KL designed and supervised the flow experiments. KD performed the RT-PCR and PCR assays for SIV RNA and DNA quantification from the lymphoid tissue biopsies. DM and JV supervised confocal- and deconvolution- microscopy and 3D image rendering and analysis. JC made the Qdot 655 multimers. CO generated the Gag CM9- and Tat SL8-specific T cell clones. MJM provided funding for the study and contributed to the study design. LJP performed the primate studies. LC designed the study, provided funding for the study and led the writing of the paper. All authors contributed to critical revisions of the paper and have approved the final manuscript.
